# Neurological sequelae of long COVID: a comprehensive review of diagnostic imaging, underlying mechanisms, and potential therapeutics

**DOI:** 10.3389/fneur.2024.1465787

**Published:** 2025-02-07

**Authors:** Grant McGee Talkington, Paresh Kolluru, Timothy E. Gressett, Saifudeen Ismael, Umar Meenakshi, Mariana Acquarone, Rebecca J. Solch-Ottaiano, Amanda White, Blake Ouvrier, Kristina Paré, Nicholas Parker, Amanda Watters, Nabeela Siddeeque, Brooke Sullivan, Nilesh Ganguli, Victor Calero-Hernandez, Gregory Hall, Michele Longo, Gregory J. Bix

**Affiliations:** ^1^Department of Neurosurgery, Clinical Neuroscience Research Center, Tulane University School of Medicine, New Orleans, LA, United States; ^2^Tulane Brain Institute, Tulane University, New Orleans, LA, United States; ^3^Department of Neurology, Tulane University School of Medicine, New Orleans, LA, United States; ^4^Department of Microbiology and Immunology, Tulane University School of Medicine, New Orleans, LA, United States

**Keywords:** long COVID, post-acute sequelae of COVID-19, SARS-CoV-2, neurological complication, chronic insomnia in COVID-19, post-COVID fatigue, cognitive impairment, brain fog

## Abstract

One lingering effect of the COVID-19 pandemic created by SARS-CoV-2 is the emergence of Long COVID (LC), characterized by enduring neurological sequelae affecting a significant portion of survivors. This review provides a thorough analysis of these neurological disruptions with respect to cognitive dysfunction, which broadly manifest as chronic insomnia, fatigue, mood dysregulation, and cognitive impairments with respect to cognitive dysfunction. Furthermore, we characterize how diagnostic tools such as PET, MRI, EEG, and ultrasonography provide critical insight into subtle neurological anomalies that may mechanistically explain the Long COVID disease phenotype. In this review, we explore the mechanistic hypotheses of these neurological changes, which describe CNS invasion, neuroinflammation, blood-brain barrier disruption, and gut-brain axis dysregulation, along with the novel vascular disruption hypothesis that highlights endothelial dysfunction and hypoperfusion as a core underlying mechanism. We lastly evaluate the clinical treatment landscape, scrutinizing the efficacy of various therapeutic strategies ranging from antivirals to anti-inflammatory agents in mitigating the multifaceted symptoms of LC.

## 1 Introduction

The onset of the coronavirus disease 2019 (COVID-19) pandemic caused by SARS-CoV-2 (Severe Acute Respiratory Syndrome Coronavirus 2) caused significant political, financial, and psychosocial interruptions on a global scale. While COVID-19 initially presented as a respiratory illness, increasing evidence demonstrates multiorgan involvement in both the acute and chronic phases (1). Although long-term complications were once thought rare, recent data from the Centers for Disease Control and Prevention (CDC) suggests that up to 6% of those infected with SARS-CoV-2 experience lasting effects (2, 3), with some studies showing elevated susceptibility to LC after SARS-CoV-2 reinfection, even among vaccinated individuals (2). However, these figures likely represent an underestimation due to the difficulty of LC diagnosis given the lack of identified clinical biomarkers and its variable constellation of symptoms and the association of asymptomatic infections with LC symptoms (4). Although the discovery of phenotypic subtypes may vastly improve diagnostic accuracy and precision for this condition moving forward (5), very little is presently known regarding the long-term effects of SARS-CoV-2 on brain function (6).

### 1.1 Current clinical criteria for diagnosis of long COVID

The CDC has officially termed the combined multiorgan impact of the Post-Acute Sequelae of COVID (PASC) as “long COVID” (LC). LC comprises the signs, symptoms, and conditions that continue for more than 28 days after a patient's initial infection (7). The landmark Researching COVID to Enhance Recovery (RECOVER) which began in 2023 established a frequency of >2.5% for symptoms to be considered clinically significant among a possible 37 symptoms (8). The most strongly correlating symptoms were post-external malaise (PEM), fatigue, brain fog, dizziness, and gastrointestinal (GI) symptoms. An additional seven symptoms, such as palpitations, erectile dysfunction, altered smell or taste, lasting cough, and chest pain, also served as the secondary components of the scoring system, being found in 2.5%−15% of patients. Correlative symptoms include dry mouth, weakness, headaches, tremors, muscle and abdominal pain, fever/sweats/chills, and sleep disturbances. Finally, the absolute frequency difference between patients with LC and uninfected individuals with these symptoms was used to establish a functional clinical severity scale of LC from 1 at the least severe to 8 at the most.

Another significant retrospective analysis cohort study that evaluated the electronic health records of over 80 million patients found nine core features of LC (9). These included breathing difficulties, fatigue/malaise, chest/throat pain, headache, abdominal symptoms, myalgia, other pain, and anxiety/depression. This study added another dimension to the time course of symptoms from before to after 90 days post-infection (6, 7, 9–11). It is now known that nearly 6–7% of patients will experience some lasting effect of SARS-CoV-2 infection (3).

### 1.2 Symptomatic and physical neurological disruptions of LC

Disruption of normal neurological function is a common denominator in LC symptomatology, ranging from mild fatigue to chronic mood and sleep dysregulation, interruption to both short- and long-term memory recall, impairment of attentional focus, and word-finding difficulty (12). Much of the current literature includes case reports or small cohort studies that assess the overlap and commonalities in clinical presentations, as reported subjectively by the patients. Here, we summarize some of these studies and the prevailing clinical picture that guides the latest understanding of LC and identify gaps in the literature where further investigation may reveal clues for improvements in current interventions.

### 1.3 Fatigue and insomnia

An average of 20–25% of patients with LC exhibit both chronic insomnia and excessive fatigue (13). Of note, compared to influenza, COVID-19 patients have a 92% increased risk of experiencing insomnia for the first time (9). The first cases of central hypersomnia, characterized by excessive daytime sleepiness, associated with SARS-CoV-2 were reported nearly three years after the initial onset of the COVID-19 pandemic (14). This discovery is remarkably salient due to its temporal correlation with COVID-19 and its prominent comorbidity with fatigue. LC reduces the quantity and quality of sleep on a nightly basis, with a decline in the quality of sleep attributed to alterations in sleep cycles (15). Patients with LC exhibit increased drowsiness (NREM Stage 1) and decreased light sleep and deep sleep time (NREM Stage 2 and 3) (15, 16).

Interestingly, the risk for any nerve, nerve root, or plexus disorder is increased by 64% in patients with COVID-19 compared to those with influenza, which has been hypothesized to be another contributing factor to sleep disturbances (9). Sleep is vital to restore bodily functions and affects cardiovascular and metabolic processes (15). Current research suggests that these alterations in non-REM sleep stages 1–3 could increase the likelihood of experiencing health issues and stress levels due to increased cortisol production (15, 17). Accordingly, the risk for any mood, anxiety, or psychotic disorder is 46% higher for patients with COVID-19 as compared to influenza and 73% higher for those with encephalopathy, reinforcing the notion that sleep disturbances and mood disorders often co-occur in LC and that SARS-CoV-2 acts in unique ways from other viruses (9). The mechanisms underlying these complications are not fully understood but are thought to involve neuroinflammation, cerebral microvascular compromise, and breakdown of the blood-brain barrier (18). Given the 85% increased risk for any outcome in patients with encephalopathy, a greater understanding of these mechanisms is critical (9).

### 1.4 Mood dysregulation

Depression, anxiety, and stress disorders, such as Post Traumatic Stress Disorder (PTSD), have increased prevalence in patients with LC. Specifically, the most frequently reported disorders by patients with LC are depression, anxiety, and PTSD (19); however, in this context, it must be noted that posttraumatic symptoms in these cohorts may not necessarily be iatrogenic. A 12-month longitudinal study of 171 COVID-19 survivors with no notable mental health history revealed a 24.6% prevalence of PTSD, with notable co-occurrence of self-reported impaired cognition at 24% (20). Additionally, symptoms of depression and anxiety were observed in both patients with acute COVID-19 symptoms and LC (21), with one study reporting new-onset symptoms of either anxiety or depression in over a third of patients with LC (22). While these symptoms are not indicative of diagnosis, they support a putative link between LC and mood disorders like depression, anxiety, and PTSD. In another retrospective study of 236,379 COVID-19 survivors, 13.66% were diagnosed with a mood disorder, of which 4.22% were receiving a first-time diagnosis. In addition, hospitalized patients had a 21% increased risk of being diagnosed with any mood disorder and a 53% increased risk for a first-time diagnosis.

For those admitted to the Intensive Care Unit (ICU), the risk for a mood disorder diagnosis increased to 22.52% (8.07% first-time), representing a 15% increased risk for any mood disorder and a 106% increased risk for a first-time diagnosis. Compared to influenza, COVID-19 survivors have an 81% increased risk of receiving a first-time mood disorder diagnosis. Of those admitted to the ICU, 22.52% received a mood disorder diagnosis (8.07% first-time). Most strikingly, the same study additionally found that patients with encephalopathy had a 73% increased risk of experiencing any mood, anxiety, or psychotic disorder and a 228% increased risk for a first-time diagnosis of these disorders (9). Additional evidence of the association between COVID-19 and mood disorders comes from a cohort study of 134 patients who were examined at a median of 113 days post-infection (range: 46-167 days), with 47.8% experiencing anxiety and 39.6% reporting a low mood. These patients were significantly more likely to experience anxiety (*p* = 0.001) and low mood (*p* = 0.031) (23). Lastly, an observational study of 1,142 COVID-19 patients at ~ seven months post-infection reports a 16.2% occurrence of self-rated anxiety symptoms and 19.7% depressive symptom (24). Thus, robust evidence seems to implicate mood dysregulation after SARS-CoV-2 infection.

### 1.5 “Brain fog”

A subset of COVID-19 patients experience headaches, dizziness, short-term memory loss, and problems with attention, information processing, and word finding (25). The World Health Organization characterizes the poor intellectual functions associated with COVID-19 as “brain fog.” Linked to memory loss, poor concentration and focus, fatigue, and slower processing speed, brain fog in patients with LC bears a remarkable resemblance to Myalgic Encephalitis/Chronic Fatigue Syndrome (ME/CFS). Additionally, cancer patients undergoing chemotherapy (especially methotrexate) experience a type of brain fog that closely resembles the brain fog in patients with LC (12, 26–28). Among a sample of 2,696 participants who met inclusion criteria for brain fog, it was found that this symptom is more prevalent among women as well as patients with respiratory problems and previous ICU admissions (29). Additional studies indicate a correlation between brain fog in patients with LC and postural orthostatic tachycardia syndrome (POTS) (30), as well as mast cell activation syndrome (MCAS) (31–35).

Moreover, MCAS has been independently linked to both POTS and LC. Furthermore, LC has been linked to Ehlers-Danlos Syndrome/Hypermobility Spectrum Disorder (EDS/HSD) (36), which has itself been linked to MCAS and POTS (30). Finally, one study noted that according to some neuropsychological measures, the emotional functioning of patients with LC tends to resemble that of patients with post-concussion syndrome, another neuroinflammatory condition manifesting with headaches, dizziness, cognitive difficulties, sleep disturbances, and emotional lability (37). While the neurological conditions which cause subjective cognitive dysfunction vary, understanding the underlying mechanism is crucial for developing therapeutic interventions (38).

### 1.6 Long-term cognitive dysfunction

LC can result in memory, attention, word finding difficulties, and executive control difficulties, disrupting many abilities fundamental to activities of daily living and professional working environments alike. As a result, recovering patients can face challenges in maintaining employment and earning an income to support themselves and their families, potentially leading to increased rates of unemployment (39). A retrospective study by the University College London reported the effects of LC in an international cohort of nearly 4,000 participants from 56 countries, demonstrating that patients with LC had difficulties returning to work after seven months due to the inherent physical and mental challenges (40). These self-reported symptoms of memory impairment, mood or behavioral disturbances, and mental fatigue may or may not correlate with imaging, neuromonitoring modalities, and neurocognitive battery findings such as altered Electroencephalogram (EEG), functional magnetic resonance imaging (fMRI), and Montreal Cognitive Assessment (MoCA) and Frontal Assessment Battery (FAB). For example, it is worth mentioning that case report studies demonstrate metabolic changes to the cingulate cortex resulting in dysregulation of mood, salient-based learning, motivation, and long-term learning habits (9). Patients with LC who are hospitalized also have a 128% increased risk of developing dementia, and in the following 6 months those admitted to the ICU have a 66% increased risk (9). For patients with encephalopathy, the risk soared to a 325% increase.

Additionally, patients with LC who were hospitalized had a 65% increased risk of experiencing an ischemic stroke and a 263% increased risk of developing Parkinsonism. Those admitted to the ICU had a 193% increased risk for ischemic stroke and a 390% increased risk for Parkinsonism (9). Interestingly, abnormal cingulate cortex metabolism, despite normal MRI findings, has been seen in a host of neurodegenerative disorders such as Alzheimer's and psychiatric illnesses such as severe refractory depression (41, 42). Damage to the neural cells involved in connections between the cingulate cortex, hippocampus, and frontal cortex may account for some subjective and objective findings in persistent cognitive dysfunction secondary to LC. There also seems to be a correlation between anosmia or hyposmia and cognitive dysfunction (43). Consistent with other neurological disorders, early intervention and rehabilitation have improved overall outcomes in these patients (44, 45).

## 2 Diagnostic tools

### 2.1 Positron emission tomography

FDG-PET imaging reveals hypometabolic patterns in nearly half of patients with LC (46). In addition, scans taken 11 months after infection reveal abnormalities and inflammation in 26% of patients with LC. This hypometabolism can be seen in the olfactory gyrus, right amygdala, hippocampus, right thalamus, brainstem, and cerebellum (48). Moreover, PET scans reveal increases in microglial activity in the brainstem and increased uptake of radioligands targeting microglial activation (47). Another study examined the temporal progression of COVID-19, from viral infection to an acute immune response with inflammation and immune cell infiltration (49). These studies support the assertion that neuroinflammation and dysfunction may be critical drivers of symptoms observed in LC. A case-series study following two patients experiencing neurological LC symptoms revealed abnormal fluorodeoxyglucose (FDG) PET findings demonstrated by hypometabolic regions within the cingulate cortex (42), with mildly impaired episodic and visuospatial memory and deficits in executive function. FDG PET revealed statistically significant hypometabolic areas localized to the anterior cingulate cortex, posterior cingulate cortex, and precuneus with unremarkable MRI results. As the cingulate gyrus is implicated in emotions, depression, memory, and decisions, these findings may reveal underlying mechanisms of LC-related neurological dysfunction (42).

### 2.2 Magnetic resonance imaging

In addition to specific functional impairments, patients with LC also have general changes in brain physiology. One study found that up to 71% of patients exhibiting symptoms after four months showed significant abnormalities in magnetic resonance imaging (MRI) (47). Among these abnormalities were white matter hyperintensities, lesions in the frontal and parietal lobes (47), and microhemorrhages that persisted up to one year after symptom onset (48). MRI also revealed reductions in gray matter thickness in the orbitofrontal cortex and parahippocampal gyrus (47, 50), brain regions important for memory processing. In addition, a three-month follow-up MRI study of COVID patients revealed increased gray matter volumes in various cerebral regions encompassing the olfactory cortices, hippocampi, and cingulate gyri (51), with the implication that abnormal changes in the olfactory system may contribute to the loss of smell commonly experienced by COVID patients.

### 2.3 Electroencephalogram

Electroencephalogram (EEG) scans have also yielded diagnostic utility in characterizing the damage caused by COVID-19 and brain function, specifically during an altered mental state characterized by confusion (52). One study found that COVID-19 patients had a lower individual alpha frequency (IAF) and a greater cortical current source density (CSD) in the bilateral frontal and central-temporal regions than non-afflicted individuals. Further connectivity analysis revealed significantly higher linear lagged connectivity (LLC), which measures the similarity between signals in the frequency domain between all the regions of interest, including bilateral frontal, central-temporal, and parieto-occipital regions (53). Another study found that in a group of individuals with both neurological symptoms and self-reported cognitive deficits exhibited abnormal EEGs at a 65% frequency rate with an additional 15% being treated for focal seizures. No significance was found between MoCA scores and EEG abnormalities, MoCA scores and fatigue severity scale scores, or EEG abnormalities and fatigue severity scale scores (54). Further investigative studies found a 61.7% frequency of altered mental status, seizure-like events (31.7%), and cardiac arrest (3.5%). They also found that 96.8% of patients exhibited abnormalities when continuous EEG monitoring was used, while only 85% exhibited abnormalities when continuous EEG monitoring was not used (52). The continued use of EEG to analyze differences in disease presentation offers a unique modality that may yield further insight into underlying mechanisms.

### 2.4 Ultrasound

Based on the observed association between blood flow and cognitive outcomes, ultrasonography has proven helpful in assessing the impacts of COVID-19. Rapid and unintrusive evaluation methods such as ultrasound may expedite patient prognoses, facilitating initiation and monitoring of therapeutic interventions. However, protocols ensuring reproducibility and scoring systems tying ultrasound results to clinical outcomes remain inadequately defined. Additional research efforts are imperative to establish standardized procedures in this regard. Given these limitations, transcranial doppler (TCD) is a safe, cost-effective, easily performed, and bedside procedure to assess cerebral blood flow in LC neurological sequelae. As cerebral blood flow is tightly regulated in healthy individuals, cerebral vasomotor reactivity (CVR) has been used as a metric to evaluate endothelial inflammation secondary to COVID-19 infection as a proxy to define chronic endothelial dysfunction. One study found that TCD effectively assessed CVR changes in a small cohort of patients with LC (10 cases and 16 controls) (55). Another study that used TCD to examine brain endothelial function shows that COVID-19 patients have impaired cerebral vasoreactivity (56). This cross-sectional observational study enrolled 49 patients diagnosed with COVID-19 exhibiting mild neurological symptoms 300 days after the acute phase of the disease. They used TCD combined with a breath-holding test (BHT), a method for assessing cerebrovascular reactivity, to assess brain endothelial function in induced hypercapnia. After the rest period and after BHT, subjects' blood flow values were statistically significantly lower in COVID-19 patients compared with the control group. Even the increase in flow velocities after BHT was lower in those infected by SARS-CoV-2 than those in the control group, indicating reduced cerebrovascular reactivity. Together, these findings consistently support the association of chronic endothelial dysfunction with LC.

Additional US abnormalities associated with LC include reduced echogenic signal of the brainstem raphe (BR) detected by transcranial sonography (TCS) (57). The cohort consisted of 70 patients, of which 28.6% (*n* = 20) had a hypoechogenic BR in the TCS examination. Intriguingly, depressive symptoms were also associated with BR alteration assessed by TCS. Depression and anxiety were present in 23% of patients six months after acute infection (58), and patients with LC with hypoechogenic raphe had significantly higher scores for depression and anxiety compared to patients with normoechogenic raphe. These associations comprise further evidence of the mood-altering effects of LC and the utility of inexpensive and rapid tools such as US to aid in diagnosis and potentially guide therapeutic strategy.

## 3 Mechanistic hypotheses underlying neurological changes of LC

### 3.1 Invasion of the central nervous system

**Figure 1:**
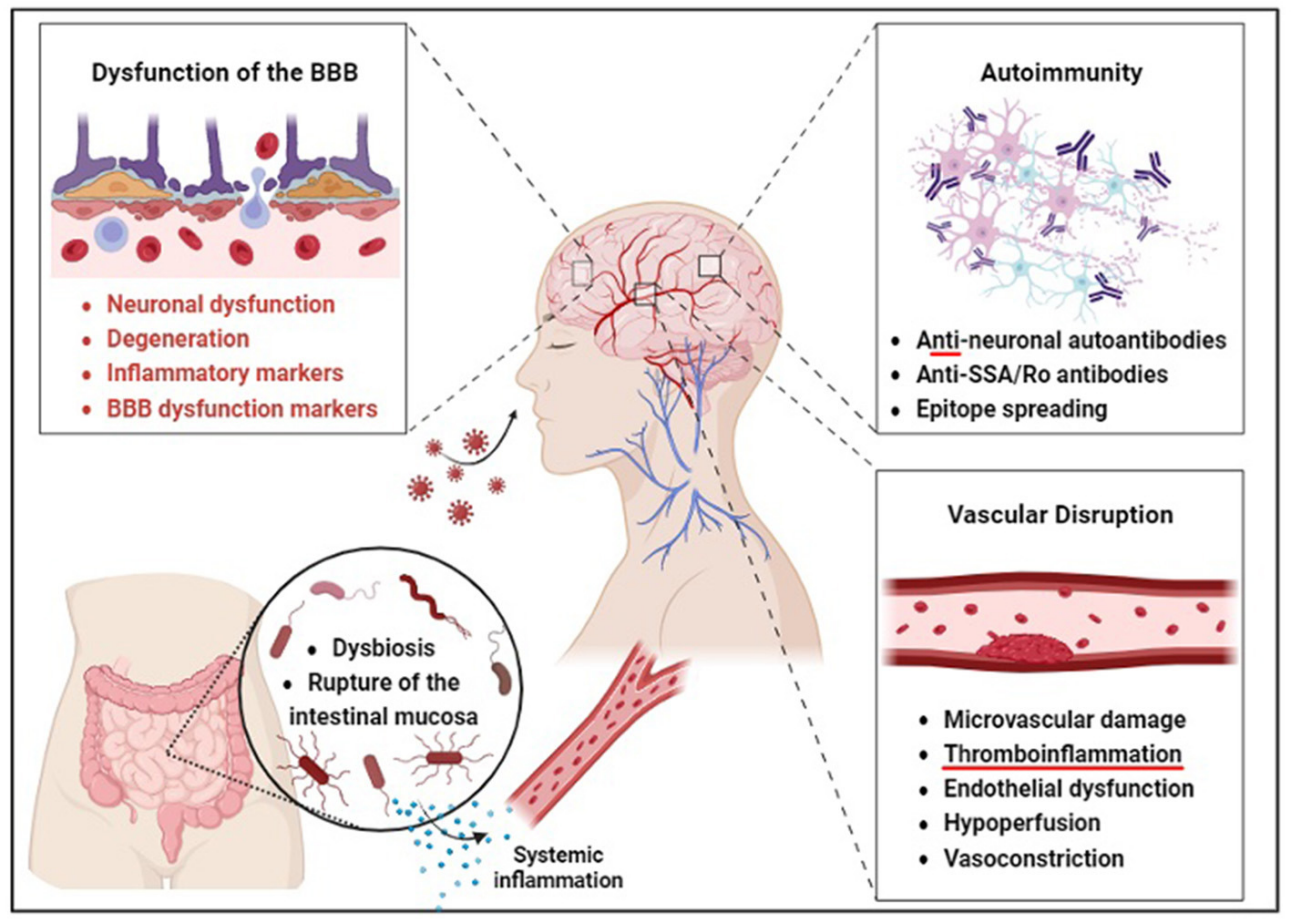
Barrier disruption may precede neurological and gut dysfunction in COVID-19 survivors. According to the direct invasion hypothesis, SARS-CoV-2 is thought to enter the brain through an aerosol-borne virus that infects lymphoid tissues and progresses to a bloodborne illness to access the CNS via peripheral nerves. The autoimmunity hypothesis is supported by the production of anti-neuronal autoantibodies and antigenic proteins of SARS-CoV-2, such as the spike protein, which may enhance immune response through somatic hypermutation inadvertent to human protein epitopes at endothelial barriers. Brain endothelial dysfunction, therefore, leads to neuronal dysfunction and degeneration. Gut microbiome composition is also significantly altered in patients with COVID-19 compared to non-COVID-19 patients, possibly due to these barrier changes. The vascular hypothesis is supported by evidence that endothelial dysfunction and hypoperfusion are central mechanisms underlying the persistent symptoms observed in these patients.

While some aerosol-borne viruses infect lymphoid tissues and progress to bloodborne illnesses via endothelial shedding, others access the CNS via peripheral nerves (62) (Figure 1). SARS-CoV-2 is known to target olfactory nerves via their surface antigen commonalities with neighboring respiratory epithelium (63). Several studies have found that acute respiratory failure may result from viral spreading to olfactory receptors in the neuroepithelium (64, 65). However, this research was conducted on human coronavirus (hCoV) rather than SARS-CoV-2 (64, 66–68). It has been hypothesized that anosmia may arise from nasal invasion and that the virus can access the CNS from that entry point (64). Available human describing spatial transcriptomic data in humans details the presence of docking receptors and viral defense genes, which support a mechanism for direct neuroinvasion (65). However, clinical evidence demonstrating CNS invasion is limited. RT-PCR testing conducted on the CSF of 578 samples during an outbreak in Lyon, France, in 2020 revealed only two slightly positive results supporting this hypothesis (69). Two confirmed cases of meningitis with SARS-CoV-2 RNA-positive CSF may offer additional insight (70, 71). The first is a 24-year-old man in Japan in 2020 displaying multiple generalized tonic-clonic seizures and nuchal rigidity with a Glasgow coma scale (GCS) of 6 and a negative nasopharyngeal RT-PCR swab test for SARS-CoV-2. The second is a 26-year-old female health worker with gastrointestinal symptoms and multiple generalized tonic-clonic seizures with a positive nasopharyngeal RT-PCR swab test. Although largely inconclusive, multiple lines of evidence implicate a hypothetical pathway for direct brain invasion. This pathway may include retrograde transport via dynein through olfactory neurons like rabies virus, viremia resulting in the crossing of the BBB via capillaries with reduced tight junction integrity as found in circumventricular organs, or hematogenous access via infected T-cells: the “Trojan horse” hypothesis (64, 72). Considering these studies, available evidence suggests that CSF testing for meningoencephalitis occurring via direct invasion of the CNS by SARS-CoV-2 may not be clinically valuable but may at least reveal some insight into cases of seizures or other symptoms indicating direct neuroinvasion. Further research is warranted to establish how SARS-CoV-2 disseminates within the CNS.

### 3.2 Autoimmunity

Another hypothetical mechanism of action for the neurological sequelae of acute and chronic COVID-19 involves anti-neuronal autoantibodies (Figure 1). This hypothesis gains credence from molecular modeling studies showing similarities between SARS-CoV-2 and human proteins. Such mimicry could lead to the accidental targeting of human proteins by antibodies generated against the virus. The process of epitope spreading further increases the risk of cross-reactivity as persistent immune activation broadens the spectrum of human epitopes available, enhancing the possibility of molecular mimicry.

The extensive inflammation and tissue damage caused by SARS-CoV-2 may activate autoimmune cells, including memory B cells, contributing to the persistence of neurological sequelae in long COVID and multiorgan involvement indicative of a maladaptive immune response. Functional autoantibodies in COVID-19 patients imply various clinical manifestations, including neurological symptoms. The heightened autoimmune response indicated by autoimmune markers like anti-SSA/Ro antibodies and antinuclear antibodies in severe COVID-19 cases (73) further supports this hypothesis. The occurrence of prothrombotic autoantibodies (74) aligns with the autoimmune contribution to COVID-19 pathology. The potential for cross-reactive antibodies to target the nervous system and cause neurological complications is explored in (75). The lack of protective immune responses in severe COVID-19 cases (76) and the immune dysregulation observed in long COVID patients (77) provides further evidence for this mechanism. The direct link between autoimmunity and neurologic manifestations is reinforced by the discovery of anti-neuronal autoantibodies in patients with COVID-19-associated neurological symptoms (78). The role of B cell responses in COVID-19, including the production of autoantibodies (79), underscores the autoimmune mechanism's potential in the disease's pathology.

Evidence has also emerged for the role of latent virus reactivation in long COVID (60). This study used comprehensive immune profiling to reveal elevated antibody responses against herpesvirus antigens, notably Epstein-Barr virus (EBV), in long COVID patients. These findings suggest a possible connection between viral reactivation and long COVID symptoms. The study also showed that antibody reactivity to specific viral antigens, including EBV components, was significantly higher in long COVID patients, indicating an altered immune response possibly related to viral reactivation or a heightened autoimmune state. Together, the findings in these studies indicate a possible mechanism of autoimmunity and the pathogenesis of LC.

### 3.3 Mast cell activation

One hypothesis describes immune dysfunction that may link LC to a previously described one. Evidence suggests that mast cells colocalize with IL1 and TNFa (80), suggesting a potential link between mast cell activation and cytokine storm observed in cases of LC. SARS-CoV-2 may trigger the rapid degranulation of mast cells during the well-characterized cytokine storm common to severe acute decompensation, inducing inflammation and ensuing chronic injury (81). This has inspired the hypothesis that the multisystem inflammatory response in long COVID could be linked to mast cell activation (82) acting as a general mediator for inflammation in different organs. Reinforcing this hypothesis, patients with long COVID symptoms resemble symptoms of those with mast cell activation syndrome (83). Considering the lack of knowledge on the pathways that can cause the pathophysiology of LC, the immunohistochemical information regarding mast cell activation may reveal crucial insight on how mast cells can potentially impact the recurrence of LC.

### 3.4 Neuroinflammation

Neuroinflammation may be another critical driver of COVID-19-related neurological dysfunction specific to long-term SARS-CoV-2 infection (84) (Figure 1). Cytokines, essential to direct and protect immune responses, can cause damage to vital organ systems when overproduced (85). Thus, this cytokine storm may be thought of as a hyperinflammatory state caused by the overproduction of cytokines, which, in turn, causes significant neuroinflammation, resulting in a vicious cycle that can lead to acute respiratory distress syndrome, the acute decompensation associated with numerous COVID-related deaths (85). This inflammation may be linked with cognitive decline and brain fog. It is known that SARS-CoV-2 is associated with neuroendothelial dysregulation due to cell death via ACE2 and transmembrane serine receptors (TMPRSS2) expressed on neurovascular endothelial cells. Viral binding of these receptors in the brain has also been linked to endothelial dysfunction and neural injury (41). Viral load and severity of symptoms of LC may include oxidative stress and hypoxia, as seen in severe respiratory compromise, which may induce neuroinflammation, microvascular inflammation, and even microthrombi, which in some cases have been linked to amyloid-like clots that are resistant to fibrinolysis (86). Ischemia induces neural cell death, which can further propagate to nearby healthy cells secondary to edematous release of neurotoxic metabolites, a process that can occur for days after the initial insult, like that seen in ischemic stroke (41, 87).

Furthermore, the cells responsible for the maintenance of the BBB, the astrocytes, express ACE2 receptors (88). Viral infection may, therefore, lead to disruption of the BBB, offering a potential pathway for the invasion of immune cells into normally immune-privileged tissue, which may explain the high incidence of autoantibodies seen in the LC patient population. This previously immune-privileged neural tissue may experience acute and long-term autoinflammatory responses related to microglial cell overactivation (41, 89). As microglia are responsible for the inflammatory response of the CNS, they are uniquely poised as potential mediators of the neurological sequelae of cytokine storms (90). Microglial responses influence neuronal activity through various direct and indirect mechanisms, including increased astrocyte reactivity, decreased oligodendrocytes, decreased myelination of axons, and decreased hippocampal neurogenesis (91). Several studies have shown increased glial fibrillary acidic protein (GFAP) reflecting astrocyte dysfunction and higher levels of inflammatory cytokines IL-6, MCP-144, and TNF-ß in neurologic patients with LC (92, 93). Evidence for this hypothesis overall appears substantial, though more research is needed to confirm the extent and effects of pathways involved.

Another pathway of note that may be strongly influenced by neuroinflammation and may play a role in the persistent nature of LC is that of vascular endothelial growth factor (VEGF). The VEGF family of signaling molecules consists of six different growth factors: VEGFA, VEGFB, VEGFC, VEGFE, and placental growth factor. Each of the VEGF family members is involved in the regulation and development of blood and lymphatic vessels. If neuroinflammation is an essential driver of LC, resulting in ischemia, cytokine storm, and endothelial dysfunction, then the VEGF pathway will likely be impacted. Specifically, high levels of VEGFA have been reported in LC (94, 95). Depending on the involvement of neuroinflammation and vascular dysregulation in LC, this upregulation may be linked to activating a common pathway shared by ischemic events and cytokines such as IL-6 and TNF-a (96).

Conversely, increases in VEGFA can also lead to an increase in inflammation through an increase in vascular permeability, allowing for easier infiltration of immune cells (97–99). Therefore, neuroinflammation could drive a positive feedback loop by impacting VEGFA, which then contributes to chronic inflammation, leading to the neurological damage and symptomology of LC (100), adding further weight to the notion of vascular dysregulation as one viable mechanistic hypothesis of LC. Despite these studies supporting the involvement of VEGFA in LC, its role and the degree of its involvement are still being investigated (95, 101).

### 3.5 Blood-brain barrier disruption

The Blood-Brain Barrier (BBB) regulates the movement of cells, molecules, or ions between the blood and the brain (102). The integrity of the BBB is regulated by various signaling pathways and transcription factors, including Wnt, Hedgehog (Hh), Sox-18, and NR2F2, all promoting junctional protein expression, suppressing inflammatory responses, and regulating the barrier (102). This critical regulation of the barrier maintains homeostasis of the CNS and prevents other coronaviruses from affecting the brain (103). However, the dysfunction of the barrier can lead to neuronal dysfunction and degeneration, as the activation of signaling pathways such as Wnt and Hh may compromise barrier integrity (102). Studies suggest LC brain fog is associated with BBB disruption in the temporal lobes (38). The sustained inflammation from the protracted immune response of LC may exert influence upon the structural and functional integrity of the BBB (38). LC brain fog is notably correlated with increases in the expression of inflammatory and BBB dysfunction markers such as Glial Fibrillary Acidic Protein (GFAP), Transforming Growth Factor Beta (TGF-B), and Interleukin-8 (IL-8) (38). Strikingly, evidence suggests that infected individuals with acute cognitive impairment have a disrupted BBB, as analyzed by the serum presence of S100ß, an astrocytic protein (38). A recent brain autopsy investigation on individuals who succumbed to COVID-19 yielded significant findings regarding matrix metalloproteinase-9 (MMP-9), which degrades collagen IV, an essential part of the basement membrane (104).

### 3.6 Gut-brain axis dysregulation

COVID-19 is increasingly associated with an ability to infect and disrupt gastrointestinal organ systems (105) (Figure 1). One study found that noteworthy alterations in the oropharyngeal microbiota-the collection of microorganisms including bacteria, viruses, and fungi in the oropharynx-in the back of the mouth of SARS-CoV-2 infected patients have altered metabolic pathways governing the metabolism of amino acids (106). While oropharyngeal microbiota is not determinative of downstream metabolomic alterations, these changes may indicate the presence of additional alterations downstream. Perturbations in amino acid homeostasis could provoke heightened intestinal inflammation mediated by ACE-2-dependent modifications in epithelial immune response (107–110). These disrupted metabolomic profiles may contribute to modifications within the immunological microenvironment, intensifying the overall pathological impact of COVID-19 (111). A two-hospital cohort study in China found that the gut microbiome composition was significantly altered in COVID patients compared to non-COVID patients, irrespective of receiving medication. Associations between gut microbiome composition and disease severity were observed among hospitalized patients. Notably, positive correlations were identified between the gut microbiome composition and circulating levels of inflammatory markers in the bloodstream of COVID-19 patients (112). The depletion of commensal bacteria such as Bacteroidaceae, Lachnospiraceae, and Ruminococcaceae and their replacement by more opportunistic pathogens like Enterococcus, Staphylococcus, Serratia, and Collinsella was also observed in these hospitalized patients, implying a significant reduction in both bacterial diversity and richness in individuals with COVID. This reduction could help to explain the increased persistence of systemic inflammation in long COVID patients through increased gut permeability leading to chronic multiorgan inflammation, including disruption of the blood-brain barrier and downstream behavioral symptoms (113, 114).

Additionally, a significant decrease was observed in the abundance of several bacteria known for producing short-chain fatty acids (SCFAs, known to be crucial to the maintenance of the integrity of the gut-blood barrier (115, 116), including the Agathobacter spp., Fusicatenibacter spp., Roseburia spp., and Ruminococcaceae genera when compared to their healthy counterparts (112).

Furthermore, one study suggests a causal link between altered gut microbiota and LC, as found in transplanted fecal samples from control patients and patients with LC in a germ-free mouse model. Animals displayed compromised lung immune responses and increased susceptibility to K. pneumoniae B31 infection, in addition to demonstrating dysbiosis-induced memory impairment resembling that found in LC (20). Of note, this is the first time that a model of LC intervened downstream of infection to replicate LC symptoms.

### 3.7 Vascular disruption

The vascular hypothesis of LC has gained considerable attention, positing that endothelial dysfunction and hypoperfusion are central mechanisms underlying the persistent symptoms observed in these patients (117, 118). Acute COVID-19 infection is known to be complicated by vascular disruption and coagulopathies, leading to diffuse intravascular coagulation (DIC) (119). DIC remains a significant cause of mortality in severe cases. This mechanism hypothesizes the binding and subsequent internalization of ACE2. Such internalization of ACE2 increases levels of the molecule it normally inactivates, angiotensin II (angII). AngII accumulation then leads to inflammation, vasoconstriction, and even fibrosis (120). M1-activated macrophages also contribute, causing endotheliitis, leading to a prothrombotic state through confirmed increases in coagulation factors (121–124). Multiple studies have identified microvascular damage and the prothrombotic effects of inflammation as standard features in patients with LC (125, 126). Some studies have detected vascular abnormalities in the form of microbleeds and decreased perfusion in patients with LC, which could contribute to cognitive deficits (127). Importantly, endothelial cells are not merely passive players but actively contribute to inflammation and coagulation, further supporting the vascular hypothesis (119, 128). Elevated markers of endothelial activation have been found in LC, suggesting ongoing vascular inflammation (120). Imaging studies, such as FDG-PET/CT, have also shown potential vascular biomarkers in patients with LC, adding another layer of evidence (129). Case reports have highlighted individual instances of vascular-related complications, such as recurrent angioedema and subacute thyroiditis, in patients with LC (130, 131). These reports add granularity to the broader findings and indicate the diversity of potential vascular issues. As such, novel recommendations have been made toward applying antithrombotic or antiplatelet therapies to target these complications (101, 118). These findings suggest that logical next steps include the establishment of viable animal models for the randomized controlled trials to test the efficacy of antithrombotic and antiplatelet medications, longitudinal studies to track the long-term vascular health of COVID-19 survivors, and mechanistic studies to unravel the molecular underpinnings of endothelial dysfunction in LC. These efforts will undoubtedly establish evidence-based clinical guidelines that could significantly improve the quality of life for patients with LC and reduce the risk of potentially fatal thromboembolic consequences.

Host genetic factors in LC represent a diverse disease entity where individual genetic variations and environmental risk factors likely play a role in its development. Evidence from a genome-wide association study (GWAS) on individuals experiencing LC, which examined data from 6,450 LC cases and 1,093,995 population controls across 24 studies conducted in 16 countries (132), revealed that individuals carrying a specific single nucleotide polymorphism (SNP) in the FOXP4 gene (rs9367106) have a higher risk of developing LC. This variant was observed to increase the expression of the FOXP4 gene in lung tissues. FOXP4, a Forkhead bOX transcription factor of subfamily P, is expressed in the lung, gut, and brain (133, 134). Previous studies have shown an association of FOX4 with an increased susceptibility to severe COVID-19 (135); despite the heightened risk of long COVID associated with severe COVID-19, this study suggested that the contribution of the FOXP4 rs9367106 polymorphism to the risk of LC was substantial and could not be only due to its association with severe COVID-19. FOXP4 gene variants could also play an important in neurologic LC, as this gene plays a crucial role in the development and maturation of the central nervous system (136, 137).

Moreover, mutations in the FOXP4 gene are associated with neurodevelopmental disorders (138), providing further support for the potential influence of FOXP4 in neurologic LC. Another study investigated SNPs from COVID-19 GWAS, revealing an association between NR1H2 and SLC6A20 gene variants and neurological complications observed in acute and LC cases (139). The NR1H2 gene encodes liver X receptor beta and has been linked to cognitive impairments in Alzheimer's disease, partly through affecting Aß accumulation and cholesterol homeostasis (140). The SLC6A20 gene encodes an amino acid transporter and is supposed to facilitate SARS-CoV-2 entry into cells (141).

## 4 Discussion

### 4.1 Pharmacotherapeutic agents for LC

The landscape of treatments for both acute and LC is rapidly evolving, with varying degrees of evidence supporting their efficacy. Significantly, increased severity of acute COVID has been associated with a higher likelihood of developing LC symptoms (2, 50). Nirmatrelvir/ritonavir has shown significant promise among acute COVID treatments, backed by a study that led to its emergency use authorization by the CDC (142). When used to treat LC, however, nirmatrelvir/ritonavir (Paxlovid) has been shown not to decrease the incidence of LC when given to vaccinated adults (143). Antiviral agents like remdesivir have also shown promise in reducing viral load and lung pathology (144). Anti-inflammatory medications, particularly corticosteroids, have been highlighted for their role in reducing the need for mechanical ventilation and shortening hospital stays (145). This suggests that effective acute treatments mitigate the risk of LC. Direct treatments for LC antihistamines like famotidine have shown efficacy in reducing a wide range of symptoms, lending credence to the importance of histamine in the severity of acute conditions, which have been correlated to chronic condition (146). Steroids like dexamethasone have been used for their anti-inflammatory and immunosuppressive properties (147). Melatonin has been suggested for treating symptoms like insomnia and fatigue (148). Early anticoagulation, particularly with aspirin, has been shown to protect the vascular endothelium and reduce thrombotic sequelae, significantly reducing 28-day in-hospital mortality (149). Modafinil has shown promise in improving fatigue and cognitive function in other conditions with fatigue and insomnia as primary symptoms, such as multiple sclerosis and narcolepsy, with a review indicating the benefits of application to LC, with the potential to improve several aspects of brain fog (150). Other treatments like ß-blockers, low-dose Naltrexone, and Intravenous Immunoglobulin are also being explored for their roles in managing symptoms like POTS, neuroinflammation (59), and immune dysfunction, although these are primarily supported by reviews (67). To characterize the landscape of existing interventions, a comprehensive guide detailing existing pharmacological treatments grouped by category has been compiled (Table 1). In summary, Well-designed, large-scale clinical trials to validate these treatments, both for acute and LC, are necessary to provide definitive and robust evidence for their use as potential therapeutics.

**Table 1:** Current and emerging therapeutic approaches for long COVID.

**Treatment**	**Mechanism of action**	**Citation**
Remdesivir	Antiviral medication (Tx LC via acute COVID)	(144)
Antihistamines (e.g. famotidine)	Antiviral properties, mast cell activation (Direct AND Tx LC via acute COVID)	(146, 151–153)
NSAIDs (incl. aspirin)	Anti-inflammatory (Tx LC via acute COVID)	(154)
Steroids (dexamethasone)	Anti-inflammatory, immunosuppressive (Direct AND Tx LC via acute COVID)	(122, 145)
Melatonin	Activator of NRF2, potential for treating insomnia, depression, fatigue, brain fog	(148)
Early anticoagulation (aspirin)	Inactivates procoagulant pathways, protects vascular endothelium	(149)
Modafinil	Increases locomotor activity (in rats), potential for treating severe fatigue	(150)
β-blockers	Used for POTS	(67)
Low-dose naltrexone	Used for neuroinflammation	(67)
Intravenous immunoglobulin	Used for immune dysfunction	(67)
BC007	Addresses autoimmunity	(2)
Anticoagulant regimens	Addresses abnormal clotting	(2)
Apheresis	Theorized for micro clots	(2)
Coenzyme Q10 and d-ribose	Supplements	(2)
Nirmatrelvir/ritonavir	Emergency use authorized antiviral	(2)
Sulodexide	For endothelial dysfunction	(2)
Probiotics	For gastrointestinal and non-gastrointestinal symptoms	(2)
Stellate ganglion block	For dysautonomia symptoms	(2)
Pycnogenol	For physiological measurements and quality of life	(2)
Metformin	Anti-inflammatory and metabolic actions	(2)
Nasal decongestant spray	Local steroid/alpha adrenergic agonist	(155)
Ivermectin	There is no specific mechanism for long COVID	(2)
Fluvoxamine	There is no specific mechanism for long COVID	(2)

This Table summarizes pharmacological and therapeutic interventions that have been studied or proposed for treating Long COVID (LC), organized by treatment category and mechanism of action. Treatments are classified based on whether they target LC directly or treat LC by addressing acute COVID-19 (Tx LC via acute COVID). Some agents have multiple mechanisms of action or applications. Treatments marked with “There is no specific mechanism for long COVID” have been studied but lack clear mechanistic evidence for LC specifically. Evidence levels vary among treatments, from well-established therapies to theoretical approaches requiring further validation.

^*^It is worth noting that the RECOVER initiative is also conducting clinical trials on solriamfetol for excessive daytime sleepiness and ivabridine for moderate POTS, but that as of the writing of this paper no results have been posted. https://trials.recovercovid.org/design

### 4.2 Impact on mental health

As discussed previously, LC can promote depression, anxiety, and stress in patients beyond what would be expected for an acute viral illness (59, 156). The psychological distress of depression, as well as anxiety caused by uncertainty about the course LC, can exacerbate existing mental health and psychiatric disorders. Cognitive symptoms such as brain fog can cause additional frustration and erode an individual's sense of self-efficacy, which can impact the subjective experience of mental health. In addition, the social isolation experienced during quarantine may contribute to feelings of loneliness and depression. The patient's quality of life is adversely affected as their symptoms constrain participation in activities that provide personal fulfillment. The patient's economic and occupational stress may be affected as the symptoms of LC can result in job loss/reduced work capacity, resulting in financial stress and decreased self-esteem and purpose. Any one of these effects may constitute stressors which may place undue burden on a patient that they may not be psychologically equipped to handle, resulting in posttraumatic symptoms that may or may not reach the clinical criteria for PTSD but still have a non-negligible impact in the long term (19). The multifaceted impact of LC on mental health underscores the necessity of comprehensive care and support for affected individuals. If patients are to make a full recovery from a prolonged disease, it is essential to address their mental health concerns. Such recovery must start with ongoing monitoring and further research into treatments and therapies for the mental effects of LC. Additionally, a thorough analysis of the continuity of holistic care is necessary to understand patients' mental state.

Given the number of perspectives and the absence of a comprehensive explanatory mechanism, a distinct pattern emerges concerning the fundamental nature of each paper: while no cause has emerged, the effects in each category can be grouped/labeled as either upstream or downstream in terms of a comprehensive etiology, confirming some LC hypotheses but not others, in a specific order. For example, psychological batteries showing reduced capacity for WM and recall memory in patients with LC appear downstream of the physical changes observed in neuroimaging studies, delineating marked hypoperfusion in the requisite brain regions. These appear upstream of microvascular injury and endothelial dysfunction, including disrupted BBB integrity, which may be downstream of altered metabolic and inflammatory signaling cascades. These signaling cascade alterations appear downstream of cytokine storms in acute cases, but the extent to which chronic illness shares a common upstream pathophysiology with such acute cases is unknown. Since viral clearance is observed in at most six weeks from even the most severe cases (157, 158) and LC can persist for years after the initial infection, the occult viral persistence/residual viral load hypothesis does not appear fully explanatory in most cases. Next, the most substantial evidence of viral particles detected in the CSF includes autopsies and two unreplicated measurements in live patients in France. In addition, COVID-19 appears to have some potential to trigger autoimmunity (159–161). Likewise, the available evidence supporting the reactivation hypothesis mechanism involving other viruses including EBV and HHV-6 appears to play a primary causative role in a subset of patients; according to a systematic review of the phenomenon, the pooled cumulative incidence estimate was calculated to be 38% for herpes simplex virus, 19% for cytomegalovirus, 45% for Epstein-Barr virus, 44% for human herpes virus 7, and not-insignificant percentages for other herpesviruses (162). Additionally, despite correlations with existing mental health conditions, the prevalence, severity, and consistency of symptoms combined with the presence of distinct imaging abnormalities do not appear to confirm a purely somatic or psychological origin.

One intriguing, uniting trend among these various hypotheses of immune dysregulation, endothelial dysregulation, BBB disruption, and coagulation activation is that they are all involved in inflammatory processes (156), which is in turn upstream of only one remaining hypothesis that could explain all the rest of these symptoms persisting for months after viral clearance: gut dysbiosis. SARS-CoV-2 is known to induce dysbiosis via binding to and downregulating ACE2R in the gut, which also downregulates the tightly linked B0AT organic anion transport, a known key modulator of the gut microbiome (105, 163, 164). Dysbiosis is known to cause reductions in short-chain fatty acid production and gut-tight junction integrity, allowing bacterial toxin lipopolysaccharide (LPS) to enter the bloodstream. Dysbiosis reduces short-chain fatty acid production and tight gut junction integrity, allowing bacterial toxin lipopolysaccharide (LPS) to enter the bloodstream. Reductions in SFCAs and increases in LPS have been linked to cognitive symptoms with similar profiles to LC (165, 166). These, in turn, are known to activate M1 phenotype macrophages, which release inflammatory cytokines such as TNF-a and IL-1B, found in high levels in LC patient blood, which in turn causes vascular inflammation in LC, which could lead to the hypoperfusion observed on neuroimaging studies (167).

Because many of these features, including dysbiosis, are shared by ME/CFS, which has long drawn attention for its marked resemblance to LC (61), it becomes increasingly noteworthy that ME/CFS has been implicated as a post-viral condition, including influenza pandemics (168) and the original SARS outbreak (169). In light of this, the words of Komaroff and Lipkin (170) appear to have accurately characterized the similarities of these conditions to the extent that they continue to predict findings with high accuracy. One last piece of evidence confirming the possible role of this mechanism as a leading candidate is the satisfaction of Koch's postulates by Almeida et al. (171), which successfully replicated cognitive LC symptoms in animal models via fecal transplants from confirmed patients with LC. Of note, according to this the ME/CFS correlation hypothesis should also predict that the same microbial alterations will be found in ME/CFS patients, and indeed, they are (172).

Finally, beyond microbiome disruptions affecting brain health, evidence suggests that ischemic brain injuries may cause rapidly altered microbiomes (173, 174), completing a vicious cycle of gut-brain disruption. Such a positive feedback loop may help explain the persistence of such disruptions in the gut and brain.

Beyond its similarities to ME/CFS, LC is also characterized by a unique signature of fibrinolysis-resistant microclots (175, 176) that can reach 200 um in diameter, sufficient to contribute to neuronal sequelae which may cause injuries such as those observed on both neuroimaging results and cognitive tests. These microclots have been shown to form via the interaction between two things, spike protein and fibrinogen (176, 177), but they probably need four: spike protein, fibrinogen, serum amyloid A, and the envelope protein, which simulations demonstrate interacts with serum amyloid A via its SK9 segment to stabilize the fibril formation (178). This additional hypothesis would explain how spike protein may be directly implicated in LC coagulopathy found in patients with SARS-CoV-2 infection, expressing all its proteins, but not patients with only the mRNA vaccines expressing only the spike protein. Further testing may conclusively demonstrate the proportion of neurological sequelae, which may be attributed to this mechanism via animal testing with an mRNA vaccine, which also expresses envelope protein, resulting in the recapitulation of LC neuronal pathology.

These similarities provide a considerable launching point for the investigation of therapeutics targeting neuroendothelial integrity, neuroplasticity, and viral load reduction, as well as mitigating auto-inflammatory activation and inflammatory immune overactivation. Taken together, they also offer an opportunity for unique insights into the relationship between the brain and mind by linking neurological and psychiatric alterations following post-viral syndromes, including LC. For example, each of these interactions appears fundamentally linked to the severity of vascular disruption, leading to cognitive disruption, which then leads to depression in the cognitive model, implying multiple inextricable cycles of cellular mechanisms influencing qualia and vice versa (179). Although the full extent of the mechanisms by which SARS-CoV-2 instigates acute and chronic neuroinflammatory responses remains unknown, future studies using tailored animal models to the vascular and immunogenic features of SARS-CoV-2 viral infection may prove crucial. The findings of the present review indicate that the subsequent weaving of such translational findings into accurate characterization of the clinical disorder will require imaging studies as a crucial link between molecular and functional clinical evaluations.

## 5 Methods

Relevant search terms were concatenated into a boolean string designed to capture all relevant studies, as follows: (“Long COVID” OR “Post-COVID condition” OR “Post-acute sequelae of COVID-19” OR “PASC” OR “Post-COVID syndrome” OR “Chronic COVID”) AND (“brain fog” OR “cognitive impairment” OR “neurological” OR “blood-brain barrier” OR “inflammation”). Four databases were queried: PubMed, Scopus, Embase, and Web of Science. 1,831 results returned from PubMed, 10,161 results returned from Embase, 2,463 results returned from Scopus, and 1,733 results returned from Web of Science. 9,481 Duplicates were removed, leaving 6,707 individual articles remaining. 6,527 articles were eliminated based on title and abstract screening for relevance to neurological manifestations of Long COVID, mechanistic studies of brain involvement, diagnostic approaches, or novelty. Of the remaining 180 articles selected for full-text review, we focused on those that provided substantive insights into pathophysiological mechanisms, presented significant clinical findings, or offered novel therapeutic approaches. We particularly sought articles that integrated multiple aspects of Long COVID's neurological manifestations or proposed testable mechanistic hypotheses. Studies were evaluated for their contribution to understanding the complex interactions between vascular, inflammatory, and neurological systems in Long COVID, with special attention to work that could help explain the persistence of symptoms after viral clearance. This approach allowed us to synthesize current knowledge while identifying promising directions for future research.

## 6 Perspectives

### 6.1 Evolution of long COVID research

The recognition of Long COVID as both a condition and a term for said condition emerged from patient advocacy in early 2020, when individuals reported persistent symptoms months after acute SARS-CoV-2 infection. Initial research focused primarily on symptom characterization and prevalence. Early studies hampered by lack of standardized definitions and diagnostic criteria. The field progressed from purely observational studies to mechanistic investigations, revealing similarities with other post-viral syndromes like ME/CFS and establishing the multi-system nature of the condition. This evolution mirrors our understanding of other post-viral syndromes, but has occurred at an unprecedented pace due to the global scale of the pandemic and rapid mobilization of research resources.

### 6.2 Current state and contribution

This review synthesizes emerging evidence that Long COVID's neurological manifestations arise from interconnected pathophysiological mechanisms rather than a single cause. Our analysis suggests that vascular dysfunction, neuroinflammation, and gut-brain axis disruption create self-sustaining feedback loops that maintain chronic symptoms. Beyond these, This represents a shift from earlier, simpler models of persistent viral infection or isolated autoimmune responses. By integrating evidence from multiple diagnostic modalities and mechanistic studies, we've shown how various hypothesized mechanisms may interact to create distinct patient phenotypes. This new framework helps explain both the diversity of symptoms and the resistance to single-target therapeutic approaches.

The striking similarities between Long COVID and ME/CFS symptoms, with only four symptoms previously considered unique to ME/CFS—motor disturbances, tinnitus/double vision, lymph node pain, and sensitivity to chemicals, foods, medications, or odors—now being reported among Long COVID patients in the results from the NINDS RECOVER study, strongly suggest that both conditions may represent variations of a common post-viral pathophysiological process. The few distinct features of Long COVID—such as specific olfactory and gustatory dysfunction and particular dermatological changes—likely reflect SARS-CoV-2′s unique tissue tropism rather than fundamentally different mechanisms of illness. This extensive symptom overlap carries immediate clinical implications: ME/CFS treatment strategies may cautiously inform Long COVID management, especially given that shared symptoms like fatigue, sensory sensitivity, and autonomic dysfunction significantly impair quality of life. Recent developments in animal models and studies using fecal microbiota transfer may further open avenues to investigate and potentially treat both syndromes by targeting underlying microbial or immune-based pathways. Such methods may include prebiotic, probiotic, and dietary interventions, which may also confer cardiovascular, and consequently prophylactic, benefits (180).

In addition, the convergence of evidence detailing significant overlap of long COVID's neurological sequelae with those of vascular dementia may point toward a common underlying mechanism of persistent vascular inflammation, potentially suggesting future clinical directions involving the exploration of existing vascular dementia treatments for long COVID. Furthermore, investigations revealing the propensity for SARS-CoV-2 to form aberrant microclots via spike protein interactions with fibrinogen point to a uniquely potent thromboinflammatory mechanism underlying long COVID which may help to distinguish it among post-viral syndromes (or ME/CFS). This could explain its singular severity while also supporting the possibility of thrombolytic or antiplatelet therapies for long COVID prophylaxis. Although existing analysis of aspirin for such purposes has yielded mixed results, such mechanistic insights suggest that further investigation including randomized, double-blinded, controlled trials for drugs targeting such pathways is promising. The next most important discovery may be that of the ideal stage at which interruption of the thromboinflammatory cascade leading to the microinfarcts and microhemorrhages observed in severe long COVID.

This shared clinical phenotype could drive new, unified approaches to addressing post-viral syndromes more broadly and, importantly, help validate the experiences of ME/CFS patients who have long faced clinical skepticism.

### 6.3 Future directions

The field must now advance along several critical paths. First, greater understanding of the mechanisms underlying the neurological sequelae of long COVID is essential to the development of effective treatment, prophylaxis, and education of the risks. This will require validation of animals to ensure accurate recapitulation of not only symptoms but also the underlying mechanisms to be studied. Thus, maximum fidelity of animal models to the observed clinical condition will be necessary for the further elucidation of resultant brain changes, especially along temporal and spatial axes. Within the mechanism underlying both acute and chronic brain changes associated with altered mental status, discovery of the key steps of said mechanism responsible for the prolonged state of cognitive impairment observed in the chronic condition will be disproportionately impactful given the snowball effect that waves of infected patients experiencing persistent symptoms may have on the global burden of disability-adjusted life years. Therefore, following establishment of the viability of said translational models, their utility may be maximized via multiomic mapping to identify the most critical nodes in the cascading feedback loops that maintain chronic dysfunction. Only then may future therapeutics confidently target long COVID etiologies rather than symptomologies.

Within clinical settings, future research must continue to elucidate biomarkers and validate subgroup stratification toward the development of accurate and useful diagnoses. Despite the progress made toward its definition and characterization, a long journey still remains on the path to successful disambiguation of long COVID and its subtypes from their differential diagnoses. As for the treatments currently in development, further clinical phase 2 and 3 trials await even the most benign drugs already approved for other conditions, for example famotidine. Then past approval of those agents for a specific long COVID indication, further research still will be needed for the investigation of combination therapies for maximum relief of symptoms. Given the broad range of symptoms observed, it may prove unwise to put all our chips on monotherapy.

In sum, the priorities of future research in this field must at a minimum include the development of standardized diagnostic algorithms, creation of evidence-based treatment protocols, and establishment of coordinated care models. In service of effective and regular clinical guideline updates based on the latest available evidence in the field, such as Cheng et al. (181). The sheer diversity of presentations, not to mention evidence for clinical subtypes, further suggest the possible utility of more personalized therapeutic approaches based on individual patient phenotypes and predominant pathophysiological mechanisms. Success will require continued collaboration between clinicians, basic scientists, and patients, with research priorities guided by both biological insights and patient needs. As our understanding grows, we may not only better treat Long COVID but also gain insights into other post-viral syndromes and chronic inflammatory conditions.

## 7 Conclusion

Taken together, these findings imply that LC may be shifting the landscape of psychiatric and neurological health worldwide. Importantly, it is the latest and most debilitating cause of suffering and economic instability as measured via disability-adjusted life years (DALYs) (132). While its acute effects appear primarily respiratory, its chronic neurological symptoms prove more elusive and range from fatigue to brain fog, persistent mood disturbance, increased autoinflammatory diseases, and increased amyloid-like plaques and clots. Mounting evidence also supports a remarkable resemblance to previously characterized post-viral syndromes (61). To wit, it is unknown whether the symptom profile of LC is truly unique when compared to other post-SARS viral syndromes (86). The overlap between LC neuronal disruption and other neurocognitive disorders such as Alzheimer's, ischemic stroke, and severe depression may yield insight into shared modalities, which suggest further investigation into the bases of these conditions.

Ultimately, the best therapeutic course of action may be a recommendation of treatments targeting the primary suspected etiology of suspected subtypes on a case-by-base basis, with adjuvant therapies targeted symptomatically; for example, dexamethasone during the acute phase of COVID-19 may help mitigate LC by targeting the severity of inflammation during acute COVID-19 as a suspected etiology of multiple subtypes; then, at a later stage, modafinil may prove useful for assisting patients struggling with activities of daily living because of central hypersomnia. Where research and clinical judgement find no contraindications for multimodal therapy, integrating several avenues of such treatments may prove the best course of action.

Overall, these findings suggest a path forward in which a complete mechanistic explanation of the etiology of LC requires an understanding of this complex condition as a series of interlinked, overlapping, cyclic molecular cascades ultimately determining the cardiopulmonary, neurological, and psychological sequelae of LC.
